# Biofabrication of the osteochondral unit and its applications: Current and future directions for 3D bioprinting

**DOI:** 10.1177/20417314221133480

**Published:** 2022-11-06

**Authors:** Patricia Santos-Beato, Swati Midha, Andrew A Pitsillides, Aline Miller, Ryo Torii, Deepak M Kalaskar

**Affiliations:** 1 Biochemical Engineering Department, University College London, London, UK; 2 Kennedy Institute of Rheumatology, University of Oxford, Oxford, UK; 3 Comparative Biomedical Sciences, Royal Veterinary College, London, UK; 4 Department of Chemical Engineering, University of Manchester, Manchester, UK; 5 Department of Mechanical Engineering, University College London, London, UK; 6 Institute of Orthopaedics and Musculoskeletal Science, Division of Surgery & Interventional Science, University College London (UCL), UK

**Keywords:** Biofabrication, osteochondral unit, cartilage, bone

## Abstract

Multiple prevalent diseases, such as osteoarthritis (OA), for which there is no cure or full understanding, affect the osteochondral unit; a complex interface tissue whose architecture, mechanical nature and physiological characteristics are still yet to be successfully reproduced in vitro. Although there have been multiple tissue engineering-based approaches to recapitulate the three dimensional (3D) structural complexity of the osteochondral unit, there are various aspects that still need to be improved. This review presents the different pre-requisites necessary to develop a human osteochondral unit construct and focuses on 3D bioprinting as a promising manufacturing technique. Examples of 3D bioprinted osteochondral tissues are reviewed, focusing on the most used bioinks, chosen cell types and growth factors. Further information regarding the applications of these 3D bioprinted tissues in the fields of disease modelling, drug testing and implantation is presented. Finally, special attention is given to the limitations that currently hold back these 3D bioprinted tissues from being used as models to investigate diseases such as OA. Information regarding improvements needed in bioink development, bioreactor use, vascularisation and inclusion of additional tissues to further complete an OA disease model, are presented. Overall, this review gives an overview of the evolution in 3D bioprinting of the osteochondral unit and its applications, as well as further illustrating limitations and improvements that could be performed explicitly for disease modelling.

## Introduction

### Osteochondral unit

**Figure 1.**
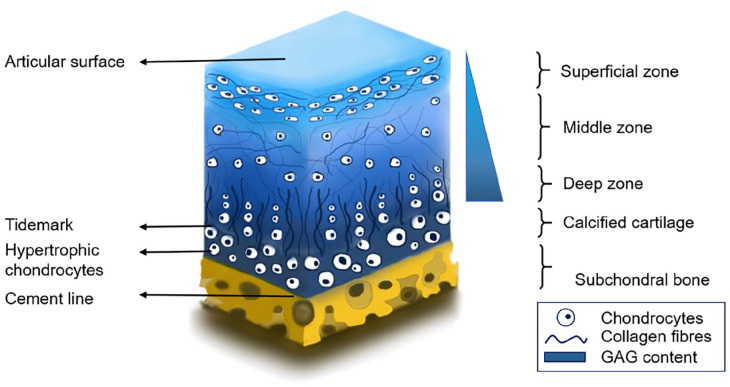
Osteochondral unit. The distribution of chondrocytes and collagen fibre alignment changes gradually from the superficial zone, where they are parallel to the articular surface, to a distribution perpendicular to the tidemark in the deep zone. Across these zones, there is also an increase in GAG content from the superficial zone towards the deep zone. GAG: glycosaminoglycan.

The osteochondral unit is formed by the intersection of hyaline cartilage and bone, presenting different areas with very distinct structures,^1^ as shown in Figure 1. In simple terms, articular cartilage is an anisotropic tissue with a composition and architecture that varies with depth. It can be divided into three zones: superficial, middle, and deep zone. These are defined by gradients in collagen deposition, proteoglycan content and collagen fibre alignment.^1^

Overall, articular cartilage is a hypocellular tissue; 2% of the total volume are chondrocytes^2^ and its extracellular matrix is 65%–85% water.^3^ This water content slightly decreases closer to the calcified cartilage. The major extracellular matrix components include type II collagen, which forms 10%–20% of the cartilage wet weight, and aggregating proteoglycans (aggrecans), which form the remaining 5%–10% of the wet weight.^4^ These proteoglycan molecules consist of negatively charged glycosaminoglycans (GAGs) covalently attached to a central protein core.^5^

When looking at the cartilage tissue in detail, further characteristics distinguish each zone. The structure and content of both collagen and proteoglycans within the extracellular matrix (ECM) change significantly across the osteochondral unit. The orientation and alignment of the collagen fibres change with depth across the articular cartilage and differ across joint locations. In the superficial zone, there is a layer of dense collagen fibres that are oriented parallel to the cartilage surface. The reduced friction and smoothness of this surface are due to these collagen fibres in addition to prevalent proteoglycans such as lubricin^6^ and synovial fluid constituents.^7^ The middle or transition zone presents an anisotropic orientation of collagen fibres with a tendency to be oblique with respect to the articular surface.^8,9^ It is also in this zone where the highest levels of the GAG chondroitin sulphate are observed.^5^ This transition zone leads to the deep or radial zone, where these collagen fibres have a radial or perpendicular orientation to the bone surface,^10^ the highest levels of the GAG molecule, keratan sulphate are present,^5^ and there is a columnar alignment of chondrocytes.

The tidemark zone is the junction of uncalcified and calcified cartilage. Collagen fibres in the radial zone extend through the tidemark into the calcified cartilage.^11^ This calcified cartilage presents 20% less dry weight collagen type II than hyaline cartilage, as well as an approximate 65% dry weight of hydroxyapatite^12^ and higher calcium content than its adjacent bone.^13^ It is connected to the subchondral bone plate and the deeper underlying trabeculae. The subchondral plate varies in thickness depending on the biomechanical forces that it is subjected to, hence changing according to joint geometry and location, age, weight, and exercise.^10^

It is throughout this tissue structure, as well as across the whole joint, that the progressive stages of diseases such as osteoarthritis (OA) are observed.

### Osteoarthritis (OA)

The osteochondral tissue can be subjected to multiple diseases such as OA, rheumatoid arthritis, osteochondritis dissecans, and additional post-traumatic injuries. However, OA is the most prevalent joint disease, which affects approximately one in 10 adults in the UK^14^ and 54.4 million adults in the US.^15^ The existing treatment options are only able to provide symptomatic relief, instead of a cure. The prevalence of OA increases with age affecting 50% of people above the age of 75, according to the National Institute of Health and Clinical Excellence (NICE).^16^ This represents an immense socio-economic challenge, with the increase in the percentage of ageing population.^17^ The estimated cost of treatment ranges between $3.4–13.2 billion per year in the US,^18^ and £10.2 billion in the UK according to the NHS.^19^

OA is a disease that affects the whole joint,^20^ it is mechanically induced, and both genetic and acquired factors contribute to its development.^21^ Although it affects the joint as a whole, the current pathophysiological models focus on the articular cartilage and subchondral bone as areas of particular interest.^20^ This is due to both cartilage and bone receiving and dissipating the stresses associated with movement and loading, which challenges biomechanically in a continuous manner.^20^

**Figure 2.**
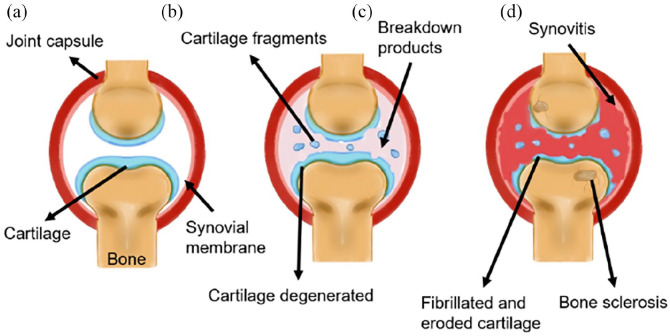
Schematic diagram of OA progression. (a) Normal healthy joint, (b) Early OA, showing degeneration of the cartilage and appearance of breakdown products in the synovial fluid, and (c) Late OA, showing cartilage loss, bone sclerosis formation, and synovitis. The amount of breakdown products in the synovial fluid increases dramatically. OA: osteoarthritis.

In simple terms, three progressive stages of OA have been characterised: stage I, proteolytic breakdown of the cartilage matrix; stage II, fibrillation and erosion of the cartilage surface and release of breakdown products into the synovial fluid; stage III, synovial inflammation as breakdown products are phagocytized by synovial cells which leads to the production of inflammatory cytokines and proteases.^22^ A schematic diagram of this progression is shown in Figure 2.

The initiation and progression of OA involves multiple tissues such as cartilage, synovium, bone, and bone marrow as well as menisci, ligaments, muscles, and neural tissues. These maintain joint stability, balance, and proprioception, ensuring homeostasis at the organ, tissue, and molecular level (Brandt et al 2006). At the cellular level, OA is first observed with cell proliferation and enhanced matrix remodelling in bone and cartilage.^20^ Synthesis of matrix molecules is increased by articular chondrocytes, as well as the production of proinflammatory cytokines such as interleukin (IL)-1 and tissue destructive enzymes such as matrix metalloproteinases (MMPs), which enhance matrix destruction. The loss of the extracellular matrix leads to a change in the articular chondrocyte phenotype, making them hypertrophic. This is followed by matrix calcification around the chondrocytes, leading to thinning of the cartilage surface and shifting of the tidemark upwards.^20^ Subchondral bone sclerosis happens simultaneously, thickening the cortical plate, generating high bone turnover, and forming osteophytes at the outer edges of the joint.^23^

Biomechanically, there is a fast formation of bone leading to the thickening of the subchondral bone. However, this increased bone formation is not accompanied by fast mineralisation.^20^ As reviewed by Lories and Luyten, this new bone is thicker but presents greater compliance and is less resistant than the thinner subchondral bone.^20^ Additionally, bone attritions are associated with both cartilage loss^24^ and bone marrow lesions occurrence.^25^ Although the causes of these symptoms are yet not fully understood, physical changes in both bone and cartilage add to the stiffening of osteoarthritic joints.

Finally, the molecular crosstalk between the two tissues is enhanced as OA progresses. Some microchannels go from the subchondral bone into the calcified and uncalcified cartilage.^26,27,28^ These channels become more abundant in the joints of patients with OA and rheumatoid arthritis, as well as endothelial cell proliferation and high vascular density.^29^

## Key parameters in biofabrication of osteochondral tissue units

**Figure 3.**
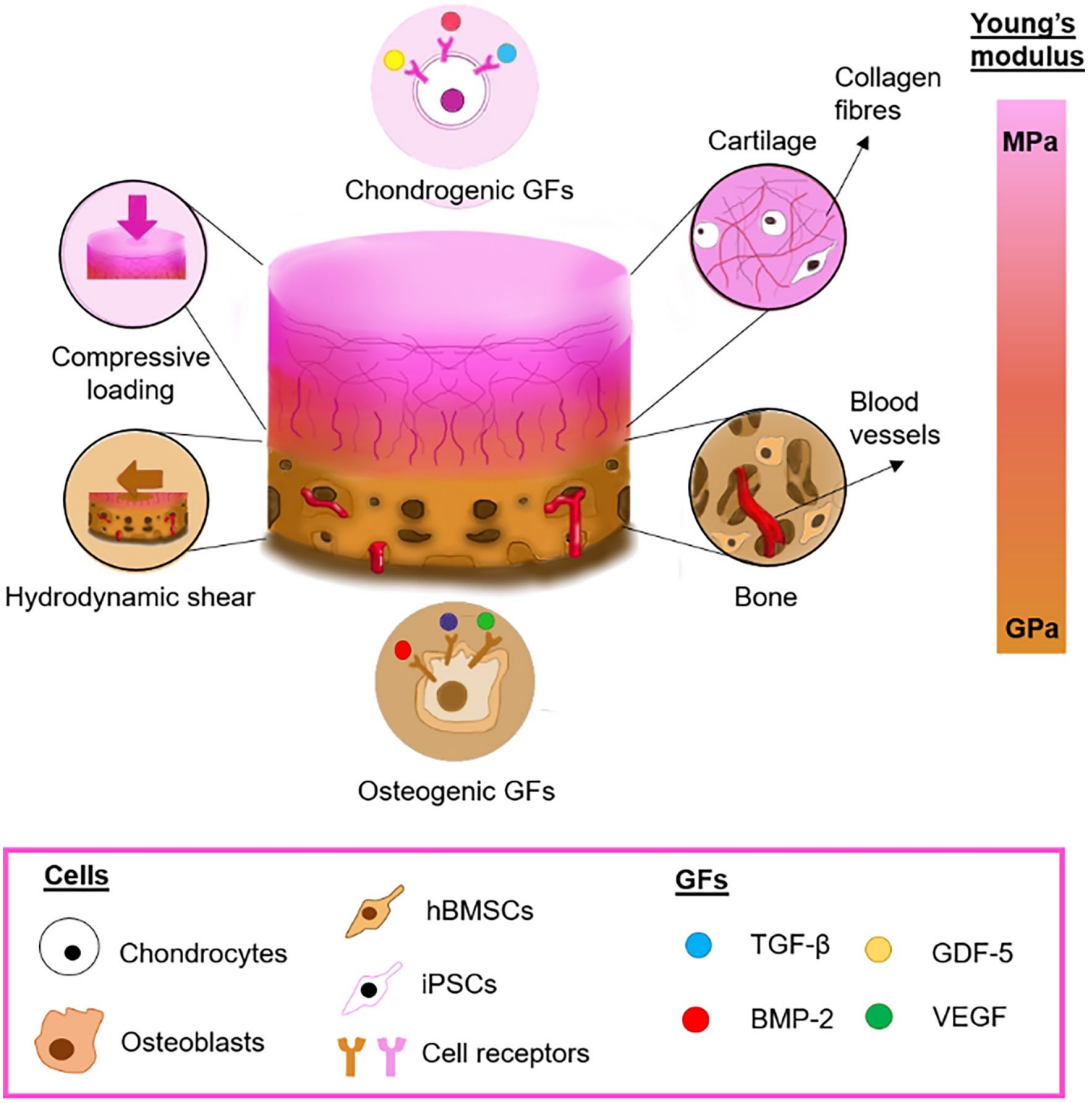
Key parameters in osteochondral unit development. hBMSCs: human Bone Marrow-derived Mesenchymal Stem Cells; BMP-2: Bone Morphogenetic Protein 2; GDF-5: Growth Differentiation Factor 5; GFs: growth factors; iPSCs: induced pluripotent stem cells; TGF-β: transforming growth factor-beta; VEGF: Vascular Endothelial Growth Factor.

A healthy osteochondral tissue, as previously explained, is extremely complex and has a specific hierarchical structure. In summary, it is composed of hyaline cartilage connected through a zone of calcified cartilage to the subchondral cortical bone (Figure 1). The latter, known as the subchondral plate, gives way to epiphyseal trabecular bone.^30^ Getting the right architecture and mechanical properties to recreate this distinct composite structure remains a major challenge in osteochondral tissue engineering. To achieve this complex multicellular system, multiple parameters such as cell choice, materials, and growth factors (GFs) need to be optimised. The incorporation of mechanical cues (shear stress and compressive loading) plays an additional key role when inducing tissue formation. A summary of these parameters is schematically presented in Figure 3. In the following sections, we will discuss each of these parameters in detail.

### Cell sources

At the cellular level, the osteochondral unit is a composite structure formed by chondrocytes, osteoblasts, and osteocytes. Chondrocytes in the cartilage section differ in shape and distribution across the cartilage depth. From the cartilage surface to the osteochondral interface, there is a gradual change from elongated chondrocytes parallel to the cartilage surface to more randomised circular-shaped cells in the middle zone,^31^ Figure 1. Closer to the osteochondral interface, chondrocytes are hypertrophic, increasing in size.^31^ These hypertrophic cells can, in vivo, become osteoblasts to form part of the bone section of the tissue, which then mature and become osteocytes.^32^ Alternatively, during in vivo osteochondral formation, bone can also replace sections of calcified cartilage and form the subchondral bone structure.^33^ To biofabricate an osteochondral construct, these three cell types with their corresponding location-dependent characteristics (size, shape, and distribution) should be present.

In vitro tissue replicas can be made using these specific cells from primary sources. However, there are limitations when using primary cells, such as presenting a finite lifespan, limited expansion capacity, and potential phenotype loss when expanded in standard two-dimensional (2D) culture conditions. Alternatively, other cell types that will mimic the tissue behaviour, such as cell lines^34,35^ could be used. These cell types will have a similar phenotype to primary cells, although they will not be the best suitable option if these constructs are meant to be used for personalised medicine.

Other options include obtaining the required cells through differentiation of stem cells (SCs), usually, human bone marrow-derived mesenchymal SCs (hBMSCs) or induced pluripotent SCs (iPSCs).^36^ The differentiation of these SCs towards osteogenic and chondrogenic lineages will require and depend upon the applied mechanical cues, such as compression or shear stress, and specific GFs added to the tissue, such as transforming GF beta (TGF-β) and bone morphogenetic protein (BMP). These two parameters are further discussed in the sections below.

### Physico-chemical parameters

At the biomechanical level, the osteochondral unit presents a gradual change of compressive moduli, which decreases from the subchondral bone region towards the cartilage surface. Human cortical bone has a Young’s modulus varying from 1 MPa^37^ to 18.6 GPa,^38^ depending upon its location. On the opposite side, cartilage presents a relatively lesser compressive modulus (0.24–1 MPa^39^). These mechanical characteristics depend on the ECM components and architecture. While cartilage is composed mostly of aggrecans and type II collagen,^40^ the bone matrix is mainly formed of type I collagen and hydroxyapatite.^41^ The secretion of these ECM components does not occur unless the physiological and external conditions are adequate.

One of the most influential factors for correct ECM deposition for the osteochondral tissue is the application of mechanical cues such as compressive and shear stresses.^42–44^ During embryonic development, joint movements ensure that the osteochondral interface is correctly formed and that chondrocytes become hypertrophic in the subchondral region.^45,46^ Further in vitro research has also shown that 3D chondrocyte cultures have a phenotype closer to in vivo behaviour when subjected to intermittent dynamic loading.^47^ In vitro 3D chondrocyte cell cultures have been subjected to a wide range of cyclic compressive strains, ranging from higher levels such as 10%^47^ to lower 5%^44^ and 1%–3%.^48^ Although the strain levels have shown to vary, the frequency of cyclic loading is maintained around 1 Hz and all have shown an increase in collagen and proteoglycan content in these tissue models compared to static conditions. Similarly, hydrodynamic shear has been shown to induce a rapid bone maturation, showing osteocyte formation and higher levels of mineralisation.^43^ However, the behaviour of osteoblasts in vitro is less defined as the hydrodynamic shear stresses applied range from millipascals^49,50^ to 1 Pa ^51,52^, lacking standardisation and optimisation for bone maturation and mineralisation in vitro. Overall, the compressive strain and hydrodynamic shear stress applied respectively to cartilage and bone still require optimisation and standardisation to achieve the required ECM formation in the osteochondral unit.

The material physico-chemical characteristics of the cell-carrying scaffolds can also determine the chondrogenic or osteogenic fate of the selected cells in vitro and in vivo. Material-dependent chondrogenesis^53,54^ and osteogenesis^55,56^ have been observed in previous studies. For example, Zheng et al. used rabbit bone marrow MSCs (rBMSCs) in collagen-based hydrogels with different compositions, comparing pure collagen hydrogels with hydrogels based of both collagen and alginate.^54^ These constructs were implanted subcutaneously in rabbits. After 8 weeks, the rBMSCs that were embedded in the hydrogels showed chondrogenic differentiation in comparison to those cells implanted with no hydrogel, which showed no chondrogenesis. Moreover, the cells embedded in the pure collagen hydrogels presented a higher level of collagen type II expression, confirming the effect that specific material compositions can have on the chondrogenesis of cells. Yang et al. also studied the material-dependent potential for both osteogenesis and chondrogenesis of multiple materials such as hydroxyapatite and tricalcium phosphate (HA/TCP), polyurethane (PU) foam, poly(lactic-co-glycolic acid)/poly(ε-caprolactone) (PLGA/PCL) and collagen type I gel.^55^ Using rat MSCs they induced chondrogenesis in vitro for 4 weeks prior to in vivo subcutaneous implantation for 8 weeks. Although all materials showed comparable levels of chondrogenesis, only HA/TCP, PU and collagen I scaffolds presented bone mature formation in vivo; showing once more the importance of material-dependent physico-chemical properties to achieve osteo and chondrogenesis.

### Growth factors (GFs)

Literature shows that for bone, cartilage, and osteochondral constructs, GFs such as TFG-β and BMP are widely used.^57^ For instance, in bone constructs, osteoinductivity is usually achieved by using BMP-2 and vascular endothelial GF (VEGF) for bone formation and vascularisation, respectively.^58^ For cartilage manufacturing, TFG-β factors, BMP-2, and growth differentiation factor 5 (GDF-5) have been used, alone or in combination, in animal^59^ and human in vitro chondrocyte cultures.^36^ Lu et al. successfully 3D bioprinted an osteochondral construct by using a combination of BMP-2 and insulin-like GF 1 (IGF-1) with human mesenchymal stromal cells.^60^ The constructs that were manufactured with GFs showed superior neonatal bone and cartilage tissue formation when tested in rabbit defects after 8 weeks of in vivo implantation, as compared to constructs without GFs.^60^ These GFs have shown to be beneficial in bone formation,^57^ vascularisation,^57^ and directed differentiation of iPSCs towards the lineage of interest.^36^ The use of TGF-β seems to be standardised, showing a concentration of 10 ng/ml when used in cartilage in vitro cultures for primary human chondrocytes^61–63^ or human MSCs.^36,64,65^ However, the use of BMP-2 in osteoblast cell culture varies dramatically from 0.5^36^ to 50 ng/ml^66^ both used in human SCs. Therefore, the spatiotemporal distribution and concentration of certain GFs, remain important parameters to be optimised.

## Use of 3D bioprinting for osteochondral unit fabrication

Conventional manufacturing techniques for cartilage and bone tissue fabrication include self-assembly,^67^ gas foaming,^68^ phase separation,^69^ freeze drying,^70^ and electrospinning.^71^ These fabrication approaches are scaffold-based strategies, where cells are loaded into either porous scaffolds, hydrogels that are then post-processed, or macro-porous scaffolds. Although these techniques have shown great potential in the field of cartilage and osteochondral regeneration, they have several disadvantages such as the lack of architectural control over the manufacture of complex tissue constructs, and poor reproducibility in terms of porosity, pore size and cell distribution. 3D bioprinting has emerged has an alternative biofabrication technique which overcomes these limitations. 3D bioprinting provides multiple advantages including reproducibility on scaffold production with control over its porosity and pore distribution required for cell survival. It additionally enables the precise control over cell distribution within the construct as well as material and growth factors, using the same manufacturing platform. The possibility to use multiple cell types and control their spatial distribution to mimic the hierarchical structure of native tissues, makes bioprinting an ideal biomanufacturing technique for the assembly of various tissue types, including the osteochondral unit.

3D bioprinting supports both cellular and acellular printing of scaffolds, which can be matured into 3D tissue structures. It is an attractive technique where hierarchically complex structures, with predefined gradients of multiple cell types, biomaterials and GFs, can be manufactured.

For osteochondral bioprinting, two main techniques are used: extrusion-based and inkjet-based. Additionally, microfluidic 3D bioprinting has been used to reproduce this tissue, although it is not widely reported. There are multiple reviews where these techniques are explained in detail,^72–75^ thus their detailed discussion is considered outside the scope of this review. In short, they differ from each other in the way they deposit the material to create 3D structures.

Extrusion-based bioprinting is a fluid dispensing system, which can be pneumatic or mechanical, controlled by an automated robotic system that extrudes and writes. Around 89% of all the published work on 3D bioprinting of in vitro osteochondral tissues between 2012 and 2022 have used this technique. It has a short manufacturing time,^72^ allows the flexibility of using multiple materials and the deposition of high cell densities within the same construct,^34,76,77^ closely resembling the physiology of native tissue.

Inkjet-based bioprinting involves depositing droplets of ink onto a bioprinting base in a precisely controlled manner.^72^ These droplets can be in the form of either single-cell suspensions or cell spheroids, as shown in a recent study by Daly and Kelly.^64^ ~11% of all published work in 3D bioprinting of in vitro osteochondral tissues between 2012 and 2022 use inkjet-based bioprinting.

Finally, microfluidics-based bioprinting, integrates microfluidic systems with traditional extrusion-based bioprinting to facilitate the hierarchical assembly of the bioprinted constructs.^75^ The bioprinter controls the flow of bioinks through microchannels using valves, which allow different components to be mixed, facilitating fine tuning of the structure and composition of the bioprinted construct.^75^ Although not widely used in bone or cartilage bioprinting, Idaszek et al. demonstrated the feasibly of this technique to manufacture an osteochondral tissue,^78^ aiming to develop a rapid drug testing platform.

### Bioinks used in osteochondral biofabrication

Bioinks, the combination of biomaterials and cells, provide a suitable physical microenvironment for cell survival, motility, and differentiation.^79^ They should exhibit high mechanical integrity and structural stability as well as demonstrate bioprintability with ease of shear thinning, rapid solidification, and formability. They should be affordable, abundant, and commercially available,^80^ to eventually translate the printed constructs to an industrial or clinical setting.

A key property of any bioink is its rheological characteristics.^81^ Some of the crucial rheological parameters to consider while designing a bioink are viscosity, yield stress, and shear thinning behaviour.^82^ Viscosity is an important factor as bioinks with low viscosity require a lower extrusion pressure for the same extrusion velocity and nozzle size than high viscosity bioinks when bioprinted. Highly viscous bioinks require high pressures to be extruded, negatively impacting cell viability^83^ as the high viscosity can increase shear stress during printing, which can lead to cell membrane rupture.^84^ Additionally, cells that survive the high stresses have shown to have abnormal behaviour post-printing, such as altering their proliferation behaviour; either by increasing their proliferation rate after experiencing moderate shear stresses or decreasing it when subjected to shear stress higher than a cell-specific threshold level.^85^ However, high viscosity bioinks present higher mechanical integrity, stability, and a high bioprinting resolution.^86^ Thus, the optimisation of physical, biological, and printing properties of these bioinks is required to achieve 3D construct stability and optimum cell survival.

No stand-alone bioink material has so far demonstrated the potential to engineer bone, cartilage, and the osteochondral unit. These complex geometries are usually achieved using multiple materials, combining different properties and acellular and cellular 3D printing. This approach enables the appropriate characteristics to be achieved to fabricate constructs that are physiologically representative of the osteochondral tissue.

A wide range of promising hydrogels are being developed to bioprint cartilage, bone, and osteochondral tissues.^87,88^ The most common bioink combinations used to bioprint osteochondral constructs are summarised in Table 1. The listed papers were selected from the Web of Science and PubMed databases after searching for the topics ‘osteochondral’ and ‘“biofabrication’ or ‘bioprinting’“. Out of the 140 results, book chapters, review papers, patents, and meetings were excluded, giving 69 results. Papers that did not include cell-laden bioprinting as a technique were excluded, excluding acellular constructs and cell top-seeding onto acellular constructs. Finally, papers that only focused on one tissue, such as only bone or cartilage were also excluded, giving a total of 24 papers between 2012 and 2022.

**Table 1.** Shows 24 papers on osteochondral bioprinting from 2012 to 2022.

Technique	Cell	Material	Outcome	Reference
Extrusion-based	hBMSCs	Alginate + silk fibroin + gelatine + phosphate	Bilayer scaffolds were manufactured through extrusion-based bioprinting of two distinct bioinks mixed with hBMSCs. For the cartilage section the alginate/silk fibroin/gelatine bioink was chosen. In the bone section, this same material combination was used having alginate phosphate-grafted to induce osteogenesis. The simple hybrid scaffold was cultured for 21 days. Alcian blue and alizarin red stains were used to measure chondrogenic and osteogenic differentiation respectively. Although immunofluorescence staining of collagen type II and osteocalcin was performed showing promising results, further quantification of these characteristic proteins and the genetic expression should be further investigated.	Joshi et al.^89^
Extrusion-based	hADSCs	Silk fibroin + PVP + nanohydroxyapatite (nHAp)	Osteochondral scaffolds were bioprinted using silk fibroin-based bioinks using PVP as bulking agent. The cartilage section was made of this bioink and chondrogenic-primed hADSCs; the bone section had osteogenic-primed hADSCs and the stated bioink with nHAp. The bioprinted construct was used as an early OA in vitro disease model. IL-1β and TNF-α cytokines were used for 7 days to induce early OA. Subsequent 7 days of culture used Celecoxib or Rhein to attenuate the OA symptoms. Chondrogenic and osteogenic markers were evaluated in healthy, diseased and recovered samples showing the ability of this platform to recreate healthy, early OA and recovered in vitro tissue behaviour. Further studies regarding later OA symptoms and the subsequent effect of the chosen drugs are necessary to fully characterise this platform as an OA in vitro model.	Singh et al.^90^
Extrusion-based	C28/I2 human chondrocyte cell line	PLA + alginate 7%wt	Hybrid scaffolds were developed through co-deposition of PLA and cell-laden alginate hydrogel. They achieved homogeneous cell distribution on the cartilage side and >75% cell viability. Cartilage specific markers were not evaluated and further investigation is required regarding the combination of the proposed acellular gradient scaffold for the bone section and the cellularized cartilage zone.	Golebiowska and Nukavarapu^91^
Extrusion-based	Primary human chondrocytes and pre-osteoblasts.	Alginate + methylcellulose+ nanoclay Laponite	Bizonal osteochondral constructs were bioprinted using a technique where GFs are delivered from the core of the printed filament towards the shell. The cartilage section contained TGF-β3 and primary human chondrocytes and the bone, BMP-2 and human pre-osteoblasts. Cartilage and bone specific markers were highly expressed. However, there were no investigations of mechanical properties.	Kilian et al.^92^
Extrusion-based	hBMSCs	Methacrylated hyaluronic acid (MeHA)/PCL; incorporating kartogening and beta-TCP	They manufactured a triphasic construct for osteochondral defect regeneration. The subchondral bone layer was made of PCL and beta-TCP; the cartilage section had MeHA with hBMSCs, PCL with kartogenin; a top anti-inflammatory layer was made of MeHA with diclofenac solution. This construct was studied in vitro and in vivo. Although it showed high expression of cartilage markers, the structural and functional properties of the scaffold are still inferior to native tissue. No data concerning the bone section was presented.	Liu et al.^93^
Extrusion-based	Human placental mesenchymal stem cells (hpMSC) + rabbit chondrocytes (RC)	(Gellan gum + methylcellulose + alginate) GAM + ceramic Li-Mg-Si particles	A biphasic construct was bioprinted using a combination of GAM, ceramic Li-Mg-Si particles and hpMSC for the bone section. The cartilage section was bioprinted on top using a combination of GAM and RC. Different concentrations of ceramic particles were tested and both bone and cartilage markers were compared. Scaffolds were only cultured for 5 days, therefore further investigations are needed.	Qin et al.^94^
Extrusion-based	Rabbit articular chondrocytes (RACs) + rBMSCs	gelMA + Silk fibroin (SF)/ gelMA + methacrylated silk fibroin (SF-MA)/ gelMA + parathyroid hormone + silk fibroin (SF-PTH)	A biphasic construct was manufactured alternating two bioinks in each section. The cartilage section was made of RACs encapsulated in gelMA-SF and gelMA-SF-PTH; whereas the bone section had rBMSCs in gelMA-SF and gelMA-SF-MA. In vitro analysis showed higher levels of collagen X and MMP13 in the bone section, showing the bioink potential to hypertrophy chondrocytes. Collagen II and aggrecan expression were higher in the cartilage section. No mechanical properties were studied in vitro. In vivo results showed osteochondral regeneration being promoted in rabbit femur defects.	Deng et al.^95^
Extrusion based	New Zealand rabbit BMSCs	Decellularised extracellular matrix (dECM) + SF + PCL	PCL was used as a bone layer frame into which bone dECM with BMSCs was printed and BMP-2. Cartilage dECM with hBMSCs and TGF-β1 was used to print the cartilage layer on top. The bioinks with GF showed higher expression levels than the control bioinks for the respective bone (col I, RUNX2, OCN, ALP) and cartilage (col II, ACAN, SOX9) markers. PCL improved the bone mechanical properties reaching 1 MPa compressive modulus in vitro. No characterisation of the interface (calcified cartilage) was performed.	Zhang et al.^96^
Extrusion-based	Foetal cartilage-derived progenitor cells	PCL + alginate	Hybrid scaffolds were manufactured using alginate and PCL as a framework. The cartilage and bone section of the scaffold were cultured in separate media using a custom made PDMS (polymethylsiloxane) coculture system. The addition of TGF-β3 and BMP-2 improved the differentiation of the progenitor cells into cartilage or bone. No compressive tests were performed.	Yu et al.^97^
Aspiration-assisted bioprinting	Human ADSCs – then differentiated into bone and cartilage spheroid	Alginate	Predifferentiated spheroids of cartilage and bone are positioned using aspiration-assisted bioprinting onto a sacrificial alginate hydrogel. This scaffold free osteochondral tissue showed the expression of the corresponding cartilage (col II, ACAN, SOX9) or osteogenic markers (RUNX2, ALP, BSP, col 1). No mechanical testing was performed.	Ayan et al.^98^
Extrusion-based and melt electrowriting (MEW)	MSCs + articular cartilage-derived chondroprogenitor cells (ACPCs)	GelMA + tricalcium phosphate + nanohydroxyapatite + pluronic + PCL	A MEW PCL mesh was used as a base for 3D printing a printable calcium phosphate-based ink (PCaP) to represent the bone section of the osteochondral plug. On top of this section an APSC-laden GelMA was infused. The osteochondral interface was made of MEW PCL and PCaP ink with no pores to ensure anchoring. These samples were cultured for 42 days in vitro, and cartilage deposition was assessed. Collagen II and GAG deposition was observed regardless in both cartilage and osteochondral constructs. There was an increase in mechanical properties in the osteochondral constructs due to the MEW PCL and PCaP section. No bone markers were assessed.	Diloksumpan et al.^99^
Extrusion-based	Porcine Adipose derived stem cells (pADSCs)	SF + PVP (polyvinylpyrrolidone) + nanohydroxyapatite	pADSCs were predifferentiated into osteogenic of chondrogenic lineages for 7 days. These were then embedded in their corresponding bioinks. Osteogenic cells were tested in two different SF-PVP inks with and without strontium doping. The chondrogenic cells were embedded in the same ink without nanohydroxyapatite. The bone section was bioprinted with the cartilage section on top. The strontium addition promoted higher osteoinductivity than the unmodified nanohydroxyapatite-based ink and showed osteocyte maturation. Cartilage specific markers were also observed with high levels of aggrecan and collagen I in chondrocytes closer to the bone section. No mechanical testing was performed.	Moses et al.^100^
Extrusion-based	Human chondrocytes (hCh)	Alginate + methylcellulose (algMC) + CPC (calcium phosphate cement)	A three zoned construct was built with a cartilage part composed of the algMC mixed with hCh bioink, a biphasic network of calcified cartilage made of the previous bioink mixed with CPC, and a CPC acellular bone section. Different combinations of these structures were tested over 21–28 days. Each section of the construct was assessed looking at cell viability, cartilage markers and gene expression or mineral concentration. Although specific tissue markers were highly expressed, the interface showed low cell viability and there was no mechanical characterisation performed.	Kilian et al.^61^
Extrusion-based	rBMSCs – induced into chondrogenic and osteogenic lineages	Alginate + gelatine + hydroxyapatite	An osteochondral biphasic scaffold was made with an alginate and gelatine mixture for the cartilage layer, and an alginate, gelatine, and hydroxyapatite gel for the bone layer. Osteogenic and chondrogenic induced rBMSCs were embedded in corresponding bioinks and 3D bioprinted on top of each other. Although these constructs were tested in vivo, in vitro testing only focused on cytotoxicity/cell viability for up to 7 days and no specific cartilage or bone markers were assessed. The in vivo assessments focused on mechanical testing and defect regeneration; both showed an increase in the experimental groups. Although this is promising, no histological markers were assessed.	Yang et al.^101^
Inkjet-based	hBMSCs	Scaffold free – spheroids using Kenzan method.	Adult hBMSCs were cultured in pellets and differentiated into their osteogenic and chondrogenic lineages for 6 weeks. The pellets were bioprinted using the Kenzan method, which enabled spheroid fusion. These individual constructs were then placed in 24 well plates with constructs of the other lineage. When adhered, they were cultured for another 4–8 weeks in a mixture of chondrogenic/osteogenic media, or only chondrogenic media. Bone and cartilage markers were assessed by histochemistry and gene expression. When cultured in media containing osteogenic factors, chondrogenic sections were converted into bone. However, when cultured in only chondrogenic media, the bone section maintained its phenotype. Although histological and gene expression tests showed high levels of cartilage and bone markers, the mechanical properties of the constructs were not tested.	Breathwaite et al.^102^
Extrusion-based	hBMSC	Alginate + GelMA + TCP microparticles	Both alginate and GelMA were mixed with TCP microparticles to assess their effect. hBMSCs were encapsulated in control and TCP bioink scaffolds and cultured in chondrogenic media for <21 days. Cell viability and calcified cartilage markers (aggrecan, collagen I, II and X) were assessed. Overall, bioink with TCP showed the highest potential to yield calcified cartilage tissue, shown in RT-qPCR gene expression results; however, no mechanical testing was performed.	Kosik-Kozioł et al.^103^
Microchannels	hBMSCs + hACs (human articular chondrocytes) Cartilage: 3:1 ratio of hMSCs : hAC Bone region: only hBMSCs	GelMa + HAMA + CS-AEMA + alginate Hyaline cartilage: 4% alginate, 6% GelMa, 4% CS-AEMA (w/v, HC bioink) Calcified cartilage: 4% alginate, 5% TCP 6% GelMa, 4%, CSAEMA, 0.5% HAMA (w/v, CC bioink)	Continuous gradient scaffold was made with the two proposed bioinks to resemble calcified cartilage and hyaline cartilage, which resembles the osteochondral interface. In vitro evaluation showed high levels of cell viability (>85%) up to 21 days as well as high secretion of ECM proteins such as aggrecan, collagen II, collagen I and collagen X. They demonstrated that a co-culture of hBMSCs and hACs showed a more hyaline phenotype than a hBMSCs monoculture. The introduction of TCP particles also ensured a hypertrophy of chondrocytes. Although promising results were observed, the crosslinking mechanism, which relies on UV light exposure, could be disadvantageous.	Idaszek et al.^78^
Inkjet-based and extrusion-based	pBMSCs + pig chondrocytes	GelMA + pluronic + PCL	A PCL scaffold was printed as a support framework for the cellular droplets of GelMA, which were alternated with hollow pluronic channels used to ensure nutrient diffusion in the bone region. The cartilage region was printed on top of these constructs. Two conditions of static and dynamic compressive loading were tested in the culture process. Constructs, which were subjected to compressive loading, showed higher levels of GAGs compared to the static conditions, whereas calcium levels in the bone region were high in both conditions. Additionally, collagen fibre orientation was assessed and, although their orientation was parallel to the surface in the superficial zone and randomly orientated in the middle zone, there is still need to mimic the perpendicular orientation in the deep zone.	Daly and Kelly^64^
Extrusion-based	hTERT-MSC line	CPC + alginate + methylcellulose	A biphasic construct was manufactured with alternating filaments of CPC and the alginate methylcellulose blended with hTERTs. These samples were cultured for up to 21 days. High cell viability was achieved throughout the culture period. Compressive strength showed to be higher than only hydrogel-based scaffolds and lower than CPC only scaffolds, reaching 2 MPa. Although not an osteochondral construct per se, the study shows the possibility of building a triphasic osteochondral construct with the three combinations of scaffolds; CPC for bone, the biphasic one presented here as calcified cartilage, and the hydrogel based one as cartilage.	Ahlfeld et al.^104^
Extrusion-based	hTMSCs	Mono CB[6] (cucurbit[6]uril + DAH-HA (1,6-diaminohexane (DAH)-conjugated hyaluronic acid (HA)) + atelocollagen + PCL	The construct was made using a PCL framework into which atelocollagen mixed with hTMSCs was used to form the bone section followed by CB[6]-HA mixed with hTMSCs to recreate cartilage. The cartilage part was also completed with DAH-HA. In vitro, cells survived and showed the correct cell specific behaviour. Bone markers (ALP, col I, OSX) as well as cartilage markers (ACAN, col II, SOX9) were assessed. The sample section with PCL, atelocollagen, hTMSCs and BMP-2 showed the highest expression of bone markers after 14 days. Section with PCL, CB[6]/DAH-HA, TGF-β and hTMSCs showed highest expression of cartilage markers after 14 days in culture. No mechanical properties were tested.	Shim et al.^105^
Extrusion-based	Rat MSCs	GelMa + gellan gum + PLA microcarriers	A biphasic construct was printed with GelMA and gellan gum mixed with rat MSCs for the cartilage region and this same mixture with PLA microcarriers for the bone region. Although they state that the construct presents no delamination in between the interfaces, there is no assessment of cartilage markers in the osteochondral section or investigation of the mechanical properties. MSCs in the bone part are shown to proliferate and mineralise in the presence of osteogenic media as well as having the expression of bone markers such as ALP and OCN.	Levato et al.^106^
Extrusion-based	MG63 human osteoblast cell line + human primary chondrocytes	Hyaluronic acid + alginate+ collagen I +PCL	A structure delimited by PCL was printed with encapsulated osteoblasts in collagen I on one side and chondrocytes encapsulated in a hyaluronic acid and alginate bioink on the other. Each cell type behaved accordingly, showing chondrogenic (col II, aggrecan, GAG) and osteogenic behaviour (RUNX2, ALP, mineralisation), respectively. Although both cell types were cultured in the same construct, the PCL delimitation inhibited interaction at the interface, creating two distinct delimited bone and cartilage constructs.	Park et al.^107^
Extrusion-based	Human primary nasal septum chondrocytes + MG63 human osteoblast cell line.	PCL + alginate	A hybrid porous construct was printed using PCL as a frame structure and alginate as a cell encapsulation hydrogel. A chondrocyte structure was printed above an osteoblast printed section. Cell viability assays showed that high cell survival. However, no other specific osteochondral markers were not tested, and mechanical characterisation was not performed.	Shim et al.^108^
Extrusion-based	hMSCs + hCh	Alginate + calcium particles	A 3D bioprinted alginate construct was cultured for 21 days in vitro and 6 weeks in vivo. Corresponding cartilage and osteoinductive behaviour were observed in vitro through the assessment of chondrogenic markers (col II, col VI) and bone markers (col I, ALP, mineralisation, osteonectin). However, interaction in between cell bodies was limited due to the dense and stiff nature of alginate. No assessment of mechanical properties was performed.	Fedorovich et al.^109^

ACAN: aggrecan; ACPCs: articular cartilage-derived chondroprogenitor stem cells; ADSCs: adipose derived stem cells; ALP: alkaline phosphatase; BMP-2: bone morphogenic protein 2; BMSCs: bone marrow derived mesenchymal stem cells; BSP: bone sialoprotein; col I: collagen type I; col II: collagen type II; CPC: calcium phosphate cement; CS-AEMA: chondroitin sulphate amino ethyl methacrylate; CSMA: methacrylated chondroitin sulphate; dECM: decellularized extracellular matrix; GelMA: Gelatine methacryloyl; GFs: growth factors; hACs: human articular chondrocytes; hADSCs: human adipose derived stem cells; HAMA: methacrylated hyaluronic acid; hBMSCs: human bone marrow-derived mesenchymal stem cells; hCh: human chondrocytes; hMSCs: Human mesenchymal stem cells; ; hpMSCs: human placental mesenchymal stem cells; hTERT-MSC: immortalised human mesenchymal stem cell line expressing human telomerase reverse transcriptase; hTMSCS: human turbinate-derived mesenchymal stromal cells; HUVEC: Human umbilical vein endothelial cell; iPSC: induced pluripotent stem cell; MeHA: methacrylated hyaluronic acid; MEW: melt electrowritting; MMP-13: matrix metalloprotease 13; MSCs: Mesenchymal Stem Cells; OCL: osteocalcin; OSX: osterix; ; pADSCs: porcine adipose derived stem cells; pBMSCs: porcine bone marrow derived mesenchymal stem cells; PcaP: printable calcium phosphate-based ink; PCL: polycaprolactone; PDMS: polymethyl siloxane; PLA: poly(lactic) acid; PVP: polyvinylrrolidone; RACs: rabbit articular chondrocytes; rBMSCs: rabbit bone marrow derived mesenchymal stem cells; RCs: rabbit chondrocytes; RUNX2: Runt-related transcription factor 2; SF: silk fibroin; TGF-β3: transforming growth factor beta 3; TCP: tricalcium phosphate; TNF-α: Tumour Necrosis Factor alpha.

**Figure 4.**
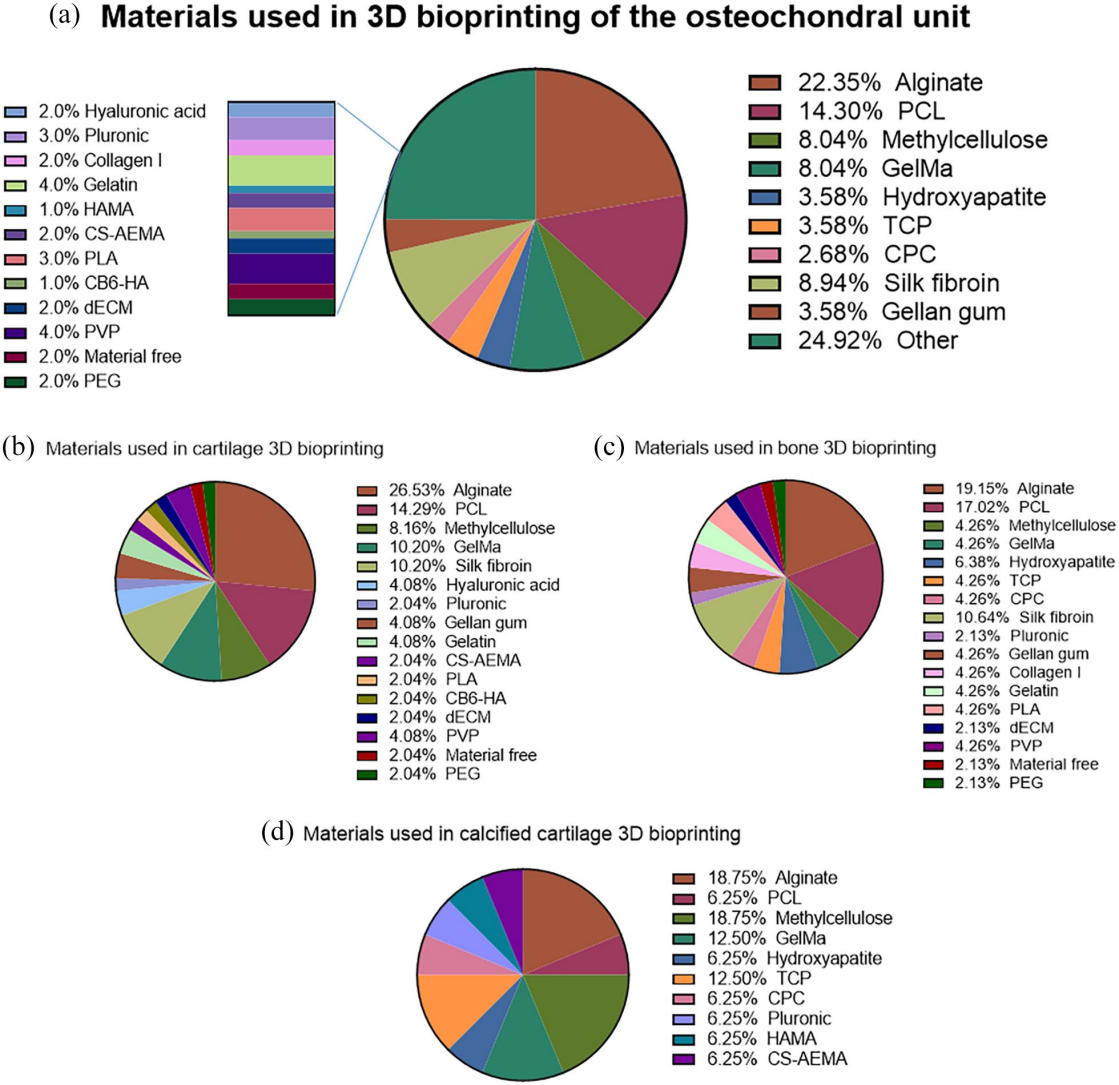
(a) Pie chart diagram showing some of the bioinks used for osteochondral unit (bone + cartilage) bioprinting. Data is based on 24 papers published between 2012 and 2022. CB6-HA (CB[6] (cucurbit[6]uril + DAH-HA (1,6-diaminohexane (DAH)-conjugated hyaluronic acid (HA)) CPC (calcium phosphate cement); CS-AEMA (chondroitin sulphate amino ethyl methacrylate); dECM (decellularised extracellular matrix); GelMA (gelatin methacryloyl); HAMA (methacrylated hyaluronic acid); PCL (polycaprolactone); PEGDMA (Poly(ethylene glycol) dimethacrylate; PLA (poly(lactic) acid); PVP (polyvinylpyrrolidone); TCP (tricalcium phosphate). (b) Bioinks used in the cartilage section of the osteochondral unit in the reviewed papers. (c) Bioinks used in the bone section of the osteochondral unit in the reviewed papers. (d) Bioinks used in the calcified cartilage section of the osteochondral unit in the reviewed papers.

**Figure 5.**
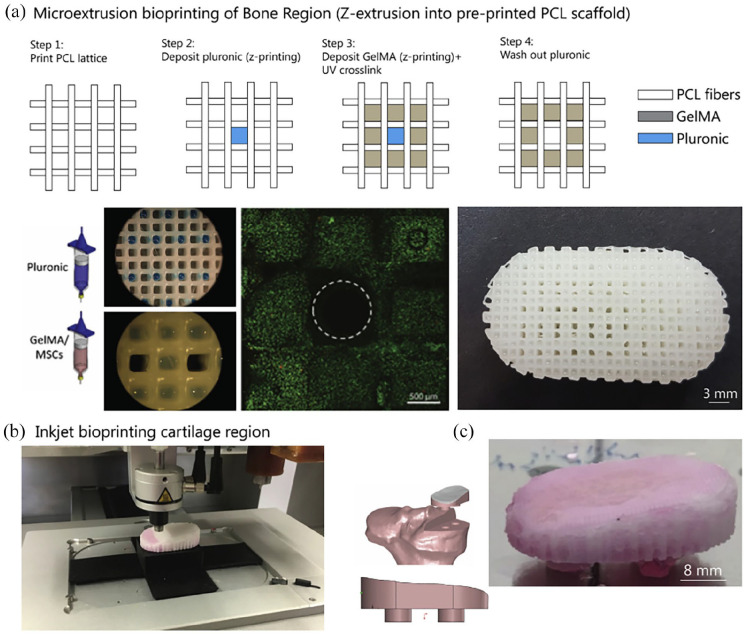
Multi-tool biopritning of osteohondral implants. (a) Microextrusion bioprinting of sacrificial pluronic component and GelMA/MSC bioink into PCL framework in bone region, live/dead analysis of MSC laden GelMA bioink including microchannels after washing out pluronic, scales 0.5 and 3 mm respectively. (b) Inkjet printing of MSC:chondrocytes suspension into microchamber system. (c) Design of unicompartmental joint prosthesis with fixation stems for use following tibial osteotomy. Macroscopic image of the tibial shaped biological joint prosthesis after 28 days of in vitro culture.

Figure 4 provides a snapshot of the most common bioinks that have been investigated in osteochondral tissue bioprinting from 2012 until 2022, specifying the materials used in each section of the osteochondral 3D bioprinted constructs. The data shows that ~22% of all published manuscripts focused on osteochondral bioprinting used alginate as bioink. This popular use is due to its instant gelation when placed in contact with ionic solutions of calcium ions (Ca^2+^), ease of use, and versatility when mixed with other biomaterials. However, alginate has poor cell attachment properties and weak mechanical properties, in the range of 3–5 kPa, which is 10^3^–10^6^ times weaker than what is needed to bioengineer bone^77,110,111^ and 10^3^ times weaker than what is needed for hyaline cartilage.^112^ Hence there is a need to explore alternative materials that present cell attachment properties, such as collagen,^113^ and better mechanical properties, like PCL,^77^ which is used in ~14% of osteochondral bioprinting manuscripts. Alginate reinforced with PCL has previously been shown to increase the compressive modulus by more than 10^3^, up to 2–3 MPa, getting closer to the desired bone modulus.^77^ A combination of cellular and acellular printing has been used to produce hybrids of hard-soft structures to reproduce the osteochondral unit.^64^ Daly and Kelly produced a PCL scaffold that was porous in the bone region and had a grid structure in the cartilage region.^64^ They used these scaffolds to print 20 wt % GelMA with encapsulated MSCs into the scaffold pores using both extrusion-based and inkjet-based bioprinting, shown in Figure 5. By using a chondrogenic medium and TGF-β3, they achieved the corresponding differentiation of these cells and the recreation of stratified cartilage in an osteochondral bioprinted construct after 28 days of culture.

Overall, both natural and synthetic bioinks have shown their advantages in 3D bioprinting of the osteochondral unit. Natural polymers such as collagen or gelatine have shown their ability to provide the bioprinted construct an architectural and functional organisation for cells. They possess necessary properties such as cytocompatibility and biodegradability. Synthetic polymers, such as PCL and PLA, also present their own advantages, presenting the ability to be modified for specific characteristics such as degradability and mechanical properties.

Additionally, there is a clear trend when recreating the bone and calcified cartilage of these osteochondral constructs, where a ceramic-based component, such as hydroxyapatite, TCP (tricalcium phosphate), or CPC (calcium phosphate cement), is chosen to facilitate osteogenesis. ~15% of the reviewed papers used one or more of these ceramic components when recreating the bone. This figure increases to 25% for the fabrication of the calcified region in the osteochondral unit. This acellular printing approach not only increases the mechanical properties of the scaffold but also improves mineralisation and osteogenic differentiation.

### Cell choice

Primary cells and cell lines have both been explored for bioprinting of bone, cartilage, and osteochondral unit. Among these, primary chondrocytes seem to be a popular choice to recreate cartilage and the cartilaginous part of the osteochondral unit. This is due to their inherent tendency to form cartilage when cultured in 3D. However, these cells experience phenotypic changes when expanded in 2D, presenting hyperthrophic and mineralisation markers.^114^ To maintain their chondrogenic phenotype, primary chondrocytes should be expanded in 3D, which makes their expansion slower in comparison to 2D culture. An alternative to primary chondrocytes is to use BMSCs or iPSCs which can be differentiated into chondrocytes with the use of GFs alone^36,115,116^ and/or in co-culture with primary chondrocyte cells.^117^

Primary cells, such as BMSCs, or human osteoblast cell lines, such as MG63, are both used to recreate bone in bone and osteochondral constructs. The choice of cell lines will only be suitable for specific applications of the resulting tissue, such as developing an OA disease model or a preliminary drug-testing model. However, if the chosen applications focused on personalised treatments, personalised drug testing applications, or implantation; primary cells will need to be considered.

Human cells are used in ~70% of osteochondral bioprinted constructs due to their relevancy in tissue mimicry. Within these human cells, primary cells are more widely used (88%) than cell lines (12%). The use of primary cells over cell lines is due to the normal morphology and cell functions that these cells present when cultured in 3D, as opposed to cell lines which can experience genotypic and phenotypic changes when passaged. These characteristics contribute towards generating a physiologically representative tissue. Moreover, within these primary cells, chondrocytes, and BMSCs are the preferred choice in osteochondral bioprinting, being used in ~29% and ~43% of cases respectively. Other cells are also used, such as adipose-derived MSC (ADSCs) and pre-osteoblasts. Primary chondrocytes, as previously stated, have an inherited tendency to form cartilage when cultured in 3D, ensuring the correct formation of this tissue. Additionally, BMSCs give the possibility of developing both bone and cartilage tissue, using a single cell type, when facilitating the correct GFs and environmental mechanical cues for their differentiation.

## Advances in improving fabrication of bone and cartilage and their implication for osteochondral tissue manufacturing

Owing to the structural complexity of the osteochondral unit, a wide range of materials can be explored to bioprint this construct and achieve the required mechanical properties (Figure 3). Incorporating a high cell density can increase the chances of obtaining a functional printed structure.^118–120^ Previously discussed 3D bioprinting techniques (extrusion-based, inkjet-based, microfluidics-based) have shown promising advances when recreating the osteochondral unit. Multiple examples of osteochondral bioprinting are shown in Table 1.

**Figure 6.**
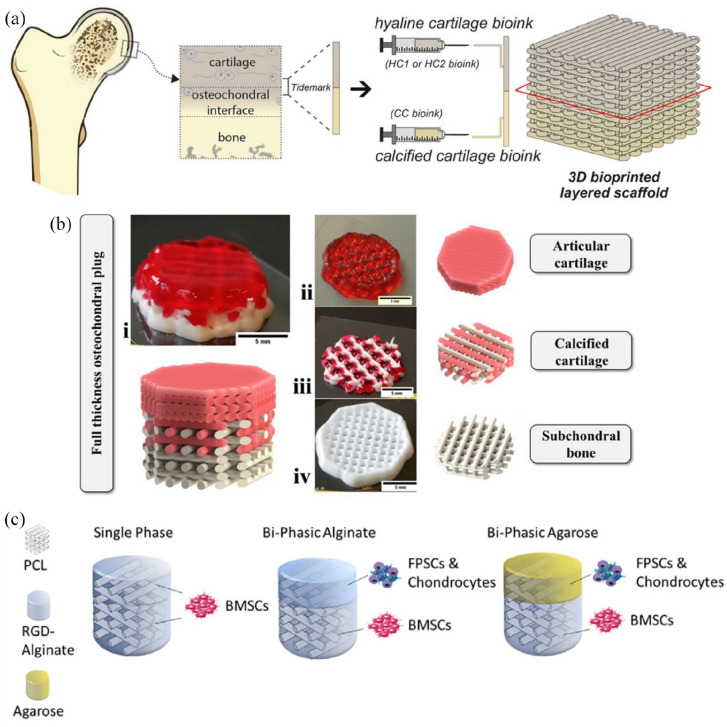
Examples of osteochondral 3D bioprinting. (a) Idaszek et al. bioprinted an osteochondral unit using two bioinks for the cartilage section, which had hBMSCs (human bone marrow-derived mesenchymal stem cells) or hACs (human articular chondrocytes) mixed with alginate, GelMA (gelatine methacryloyl) and CS-AEMA (chondroitin sulphate amino ethyl methacrylate; CSMA: methacrylated chondroitin sulphate), and for the bone section, which had the same formulation as cartilage with added TCP (tricalcium phosphate) and HAMA (methacrylated hyaluronic acid )mixed with hMSCs (human mesenchymal stem cells). Reproduced from IOP Publishing.^78^ (b) Kilian et al. built a three zoned construct with a cartilage section made of alginate and methylcellulose mixed with hCh (human chondrocytes), a middle region made of the same cartilage bioink mixed with CPC (calcium phosphate cement), and a final acellular CPC bone section. Reproduced from Scientific Reports.^61^ (c) Critchley et al produced three constructs with a PCL (polycaprolactone) internal skeleton where RGD-Alginate or Agarose was used as carriers for BMSCs, FPSCs (fat pad derived stem cells), or chondrocytes. Reproduced from ELSEVIER.^123^

The most recent examples provide insight into the evolution of osteochondral tissue bioprinting (Figure 6). Critchley et al. recently developed an osteochondral construct with multiple combinations of cellular casting and acellular printing. Three acellular materials were tested, PCL (polycaprolactone), PLA (poly(lactic acid)), and PLGA (poly(lactic-co-glycolic acid)), in combination with BMSCs and RGD-alginate, to establish a stable material that could reinforce the osteochondral construct. The study showed that PCL was stable over the 21 days of culture and increased the compressive modulus of the constructs. Structures were made using 3D printed PCL as a reinforcing internal skeleton and a mixture of alginate and agarose as cell carriers, made using agarose moulds. BMSCs were chosen for the osseous section and chondrocytes combined with fat pad-derived SCs for the cartilage section. Both the combination of acellular printing and cell-laden hydrogels show the potential of recreating this biphasic osteochondral tissue, which presents higher compressive moduli than those without the internal skeleton. Additionally, combination of multiple cell types, especially in the cartilage part, which had both SCs and chondrocytes, showed better results in terms of cellular proliferation. These approaches are currently being explored in the osteochondral bioprinting field. Simul-taneously, further techniques such as bone vascularisation and further tissue reinforcement, have been used in bone and cartilage bioprinting individually. It is worth noting that till date there are no studies which have tried to further mature 3D bioprinted osteochondral tissues in vitro using additional mechanical cues such as fluid shear stress or dynamic compressive loading. The effect that mechanical cues have on both cartilage and bone tissue maturation in vitro are well known, as previously described. It is expected that the combination of acellular and cellular printing, multiple cell types, and external mechanical cues, will be at the centre of future osteochondral biofabrication strategies.

Bone bioprinting has been enhanced by the addition of functional vascularisation. In 2020, Chiesa et al. developed a 3D bioprinted bone construct using multiple materials and techniques. Firstly, they printed an acellular scaffold of gelatine and nHA (nanohydroxyapatite) onto which hMSCs (human Mesenchymal Stem Cells) were seeded. These cells were left to differentiate for 2 weeks. Secondly, HUVECs (Human Umbilical Vein Endothelial Cells) suspended in a GelMA and fibrin hydrogel 1:1 v/v, were bioprinted into the bone scaffold macropores. After another 2 weeks, completing 4 weeks of culture, the corresponding constructs showed osteogenic differentiation of hMSCs having a functional vasculature system.^120^ The addition of vasculature in bone bioprinting not only enhances the osteogenic profile of the biofabricated construct, but also brings a more physiologically relevant in vitro bone system closer to reality.

Cartilage bioprinting has also shown improvement, as the newest examples are achieving tissue compressive moduli closer to physiological values. This is the case of Antich et al. who in 2020 were able to 3D bioprint a cartilage construct with a compression modulus close to 4 MPa after 4 weeks of culture. To develop such constructs they used a PLA acellular printed scaffold that would act as a support material; a mixture of hyaluronic acid (1%) and alginate (2%) was used as a hydrogel for suspension of human articular chondrocytes and subsequently bioprinted into the scaffold pores.^121^ Although the enhancement of mechanical properties is a key factor for recreating cartilage in vitro, additional improvements such as the recreation of cartilage anisotropic microarchitecture are necessary. Although the manufacturing of different cartilage layers has been achieved using 3D bioprinting techniques,^122^ the use of 3D bioprinting to recreate full osteochondral constructs with physiologically relevant anisotropic architecture, remains unexplored.

These recent advances give an insight into the likely future trend that osteochondral bioprinting will follow. Firstly, the combination of cellular and acellular printing enables the simultaneous use of less viscous biocompatible hydrogels, such as GelMA or hyaluronic acid, with more rigid materials that enable structural stability and microarchitectural control such as PLA or PCL.^105,107,121^ This technique combination will allow tissue constructs to achieve closer to physiological mechanical properties and microarchitecture relevance in both bone and cartilage while maintaining a high cell density deposition.

Secondly, the use of multiple human cell types appears to be a better choice when trying to translate this tissue manufacturing into the clinic and achieve better cell proliferation and tissue maturation.^123^ These cells will most likely be different cell types, enabling the formation of different tissues, such as bone and vascular tissue or SCs and chondrocytes which have shown better cartilage proliferation in a co-culture. Finally, long incubation periods of the fabricated constructs are necessary so these constructs can be used in disease modelling or drug testing.

## Applications of biofabricated osteochondral tissues

There are two main areas where the application biofabricated osteochondral tissues has been investigated; as regenerative therapies using 3D biofabricated constructs, and as in vitro disease models and drug discovery.

### Osteochondral tissues as regenerative therapy (pre-clinical bioprinted studies)

Most studies in this area are at either laboratory investigations or pre-clinical stage. Large osteochondral defects (>8 mm) of the joints require successful repair and regeneration of the articular cartilage along with the underlying subchondral bone. For this, a pre-requisite is the bioengineering of a regenerative tissue that recapitulates the structure and functional complexities of the full osteochondral unit. The biofabrication strategies (3D printing and/or 3D bioprinting) show promise in addressing this huge unmet clinical need. Common biofabrication approaches for successfully regenerating bone and cartilage units have typically used hydrogels; hydrogels, and/or polymers in combination with a ceramic phase; decellularized extracellular matrices either alone or in combination with autologous/allogenic cells; as highlighted in several reviews.^124,125^ Combination approaches to create multi-layered architectures are being employed to represent different phases (cartilage, bone and vascular) of the osteochondral constructs. However, creating such structures with distinct functionality and biomechanical compliance over the long-term is challenging.

Of the several osteochondral constructs developed, only a few of these bioprinted constructs have been tested in vivo. Idaszek et al. bioprinted layers of articular cartilage and calcified cartilage, using cellular (hMSCs and human articular chondrocytes) and photo-crosslinkable hydrogel (4% w/v alginate + 6% w/v GelMA + 4% w/v CS-AEMA + 0.5% w/v HAMA) gradients, while the underlying calcified cartilage was induced with TCP microparticles to generate a bi-phasic construct.^78^ In vivo analysis in rodent osteochondral defects showed repaired articular cartilage, rich in tenasin and collagen type II, formed within 12 weeks. While smaller animals are optimal in terms of establishing the biocompatibility of the experimental materials in vivo, they are not truly indicative of the clinical outcomes due to their marked variation with respect to human joint physiology and load.

The importance of manufacturing architecturally relevant osteochondral structures for achieving in vivo osteochondral regeneration has also been proven. Sun et al. created a gradient-structured scaffold that mimicked four distinct layers of the cartilage section, from calcified cartilage to the smooth surface. A scaffold with 4 different porosities was manufactured using PCL and populated with rabbit BM-MSCs. Although the full osteochondral architecture was not fabricated, missing the subchondral bone section, it was able to recreate the arrangement of chondrocytes in a gradient similar to native cartilage when implanted in rabbits.^122^ Other examples corroborate this important microarchitecture control,^126^ regardless of not using 3D bioprinting per se. Qiao et al. recreated a full osteochondral unit using melt-electrowriting and infusing the porous gradient structure with three different bioinks encapsulating rabbit MSCs and growth factors. These constructs recreated superficial and mid-deep cartilage sections adjacent to a subchondral bone section. They were also implanted in vivo and showed promising results in osteochondral defect regeneration.^126^

A few studies have so far implanted the bioprinted constructs in large animal osteochondral defect models, and the outcomes vary. Critchley et al. produced 3D bioprinted mechanically reinforced (PCL, PLA, and PLGA) MSC-laden alginate hydrogels to mimic the bi-phasic osteochondral morphology, wherein the overlying cartilage phase was developed using a co-culture of chondrocytes and infrapatellar fat pad derived stem/stromal cells.^123^ Following the establishment of biocompatibility and biosafety in subcutaneous implantation in nude mice, constructs were evaluated in a clinically relevant, caprine model of osteochondral defect repair. Six-months post-implantation, the constructs showed superior healing in vivo indicative of hyaline-like cartilage formation, as compared to commercial Maioregen. However, the authors indicated significant variation in the quality of neo-tissue formation, pointing towards the need for further standardisation in the design of 3D bioprinted implants.

Another study published by Mancini et al. demonstrated the osteochondral healing potential of a bi-phasic composite that represented the zonal distribution of cellular gradients (articular cartilage progenitor cells (ACPCs) with hMSCs) mixed with hyaluronic acid/poly(glycidol) hybrid hydrogel, reinforced with PCL bone anchor.^127^ A 6-month long study in an equine model resulted in the limited formation of cartilage tissue, both in the zonal and non-zonal constructs, raising serious concerns about the viability of the implanted cells and the bioresorption rate of the hydrogel in vivo. To negate the adverse effects of the materials, scaffold-free cellular spheroids are being developed for treating joint lesions. Initial studies using ADSCs spheroids in a rabbit model,^128^ as well as microtissues engineered from more advanced cell sources like iPSCs derived organoids^129^ and embryonic SCs^130^ have given encouraging initial findings, warranting further in vivo testing in large animal models to establish their long-term efficacy in clinically conformant lesion sizes.

**Figure 7.**
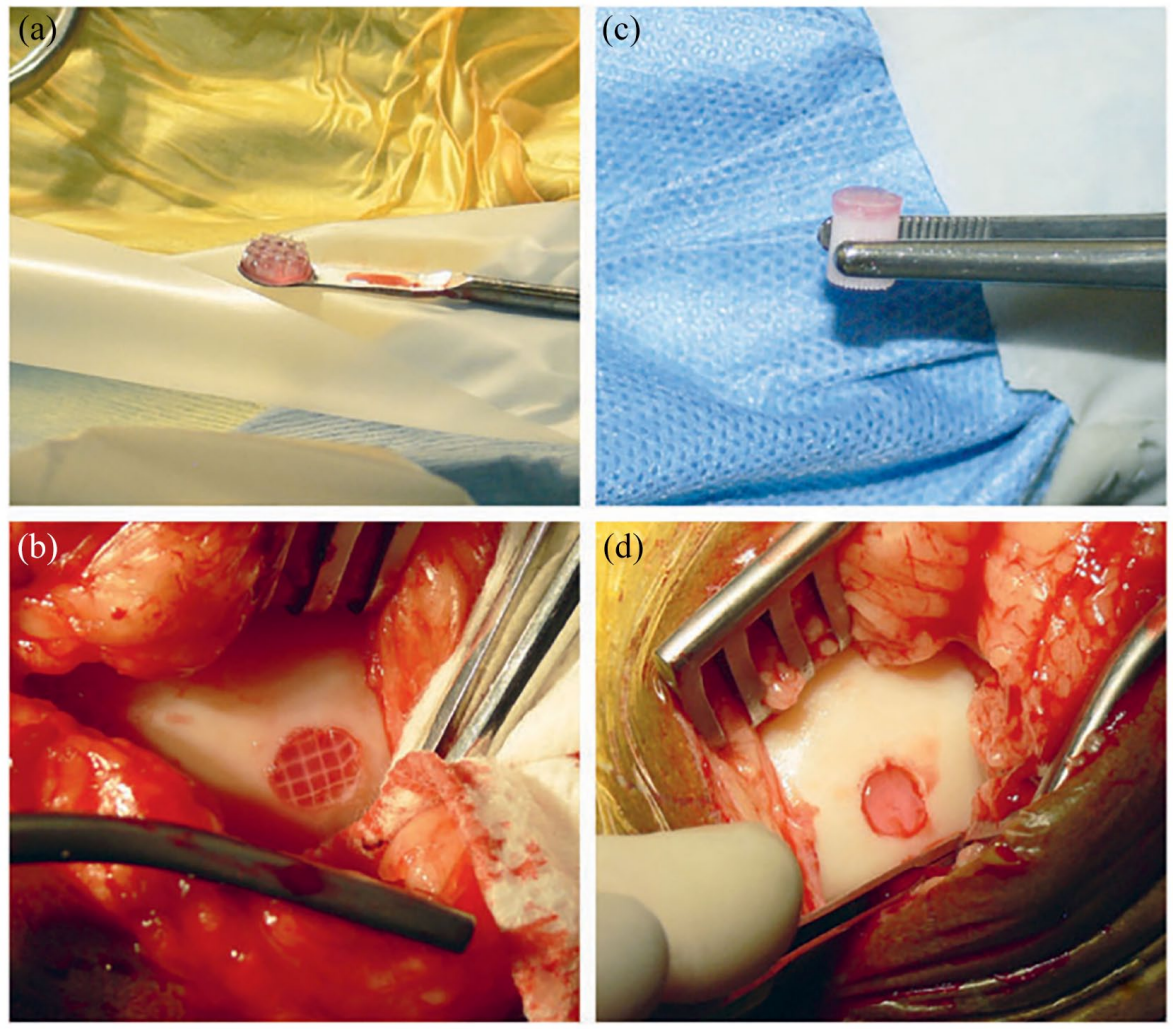
Mancini et al. Surgical implantation of materials for comparison of fixation with fibrin glue (left) versus osteal anchor (right). The reinforced hydrogel (a) was implanted in a fill-thickness chondral defect and fixated with autologous fibrin glue (b). The hydrogel with PCL osteal anchor (c) was inserted in the osteochondral defect and secured by press fit (d).^127^

Proper fixation/integration of the implants at the osteochondral injury site is a critical^131^ issue that determines the fate of the implant in the host. For this, several options are being explored, such as fibrin glue (commercial vs autologous) or osseous anchors like 3D printed PCL. Diloksumpan et al. engineered a novel implant combining bioprinting-based chondral-bone integration method using melt electrowritten PCL for the cartilage layer, which was firmly anchored with a bone component.^132^ The implants, pre-seeded with ACPCs, were primed for chondrogenic differentiation using BMP-9 for 28 days resulting in an increased expression of GAGs and collagen type II in vitro, followed by implantation of these bioengineered constructs in the stifle joints of Shetland ponies. Ex vivo analyses using biochemical and histological testing revealed minimal deposition of GAGs and collagen type II in the chondral layer of both, pre-seeded and cell-free implants, after 6 months. The failed repair outcomes were attributed to the collapse of the bone anchor, which eventually resulted in the loss of the mechanical and structural integrity of the chondral region as well. The osteal anchor made of 3D printed PCL used by the authors was validated in vivo in a horse orthotopic model previously,^131^ albeit over a short-term (4-week) only, which stresses the importance of conducting long-term in vivo studies to assess the efficacy of osteochondral repair techniques, Figure 7. This learning was echoed by several others.^131,133^

While the initial preclinical outcomes using 3D bioprinted implants show several pitfalls and roadblocks, every effort is being made to resolve these issues. With advancements in new generations of biomaterials and SC sources to improve the quality of tissue repair; technological innovations in biofabrication methods to produce stronger implants; and improvements in understanding of the OA articulation and aetiology, finding an effective treatment strategy for this rather complex tissue may not seem too farfetched.

### 3D bioprinting OA tissues for disease modelling and drug screening

Over the last few decades, the landscape of OA investigation^134^ has massively transformed with rigorous investigations using some relatable, but independent disease model systems, to address a substantial unmet need for accelerated discovery of disease-modifying OA drugs (DMOADs). Several research groups have successfully recreated osteoarthritic tissues in vitro and studied the effect generated by changing various parameters, including the addition of pro-inflammatory cytokines (namely, IL-1β, IL-6, IL-8, and tumour necrosis factor α (TNF-α), pro-catabolic mediators (MMPs 1, 3 and 13, aggrecanases, disintegrin and metalloproteinase with thrombospondin motifs – ADAMTS^135^), co-culture^136^ conditions, and external flow-induced stress or mechanical strain.^47,137^ Recapitulating such dynamic microenvironments requires stringent control over the process parameters for accurately replicating OA in vitro, which undoubtedly demands smart and sophisticated approaches. Achieving the complexity of multiple tissue types within a single construct, and achieving a clinically conformant size and function remains challenging and a key target of advanced biofabrication approaches.

Recently, Singh et al. have developed what appears to be the first 3D bioprinted osteochondral-based in vitro disease model for early OA.^90^ As previously summarised in Table 1, silk fibroin-based bioinks were used alone or combined with nHA to recreate the cartilage and bone sections of the osteochondral unit in vitro, respectively. After preconditioning hADSCs to the corresponding chondrogenic or osteogenic lineages, the cells were bioprinted and cultured in pro-inflammatory culture media for 7 days, using cytokines such as IL-1β and TNF-α. The addition of these cytokines only showed the recreation of early OA symptoms in the printed constructs, which were then partially reversed in the next 7 days of culture, through the treatment of Celecoxib or Rhein as anti-inflammatory agents. Although this approach shows promising results as a 3D bioprinted OA in vitro disease model, there are multiple aspects that require further investigation. To start with, the early onset of OA is characterised by multiple changes in the osteochondral unit, therefore the sole addition of cytokines might not be the most physiologically relevant approach to develop the early stages of this disease. Additional inputs such as mechanical loading and material-dependent stiffness changes could be included. Moreover, as it has been presented in this review, OA is a disease which affects the whole joint. Therefore, the further complexity of this model requires the addition of multiple cell types and structures to fully recreate the joint system in which OA can develop.

Independent investigations^36^ are optimising protocols for 3D bioprinting organoids using iPSCS and ESCs (embryonic stem cells) for osteochondral tissue regeneration. Dalgarno et al. developed a 3D bioprinted cell culture platform containing multiple cell types representing different regions of the human joint (osteoblasts, osteoclasts, synoviocytes, chondrocytes, immune cells) using an eight-channel cell printer in order to create a stable OA model for drug testing.^138^ While the initial pilot data demonstrated that this multi-cellular system was viable for 72 h in vitro, subsequent studies will focus on combining it with microfluidic devices to incorporate the necessary mechanical stimulations of the load-bearing joints. Thus leveraging these advances in the cellular, biomaterial, and technological domains, will together, lead to more sophisticated biofabrication strategies to create human-relevant, personalised, and reproducible OA models and provide scalable platforms for drug screening and disease investigations for translational medicine.

## Challenges and future direction

Despite the progress that has been made using 3D bioprinting to recreate bone, cartilage, and osteochondral constructs, the fabrication of a representative osteochondral unit for disease modelling using this technology remains largely unexplored. This is due to the numerous challenges and limitations that arise when reproducing a functional and enduring osteochondral tissue construct.

There are limitations associated with the 3D bioprinting process, including the bioinks and the versatility of the bioprinters themselves.^72,139^ On top of these technical limitations, there are additional issues that must be overcome to develop more representative osteochondral tissues and functional OA disease models. Three main challenges stand out: the need to standardise physico-chemical and mechanical properties of materials closer to physiological values, establish a functional vasculature system in the bone section of the construct, and the use of multiple tissue types in the osteochondral tissue-based disease model.

### Choice of bioink: Combining acellular and cellular 3D bioprinting

Current bioinks used in osteochondral bioprinting, do not mimic the necessary mechanical properties shown by natural osteochondral tissues. New material combinations must be explored to optimise the mechanical properties closer to that of native tissue. As previously stated, bone and cartilage present very different mechanical moduli. To obtain such diverse properties in one construct, multiple materials must be combined in such a way that a stable gradient forms across the middle region that ensures cohesion of both the components in the osteochondral construct. Obtaining the high mechanical modulus of bone is the biggest challenge, as high viscosity bioinks which would increase the Young’s modulus of the printed construct, could compromise the cell viability by needing high extrusion pressures. Combining materials such as PCL and alginate,^107^ or including nanohydroxyapatite^120^ in already successful bioprintable materials, have been proved to be practical strategies to enhance the mechanical properties of the printed constructs.

### Tissue maturation: Using bioreactors to improve tissue development and biomechanically model OA

**Figure 8.**
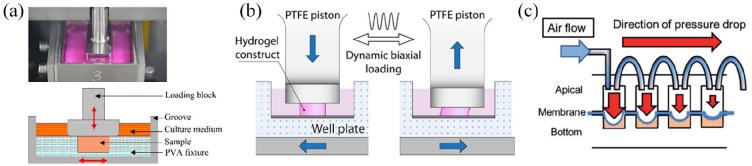
Examples of compression bioreactors used for enhancing cartilage properties and generating OA disease models. (a) Compressive bioreactor which can generate compressive load vertically and shear stress through the horizontal movement of the platform. Reproduced from Springer.^142^ (b) Compressive bioreactor which can perform compression and shear on the samples through the vertical displacement of the piston and horizontal plate movement. Reproduced from Scientific Reports.^62^ (c) Compression device which relies on air pressure to perform compression over the samples using flexible membranes. This device was used by Young et al. to study the effect that mechanical loading has on OA-like chondrocytes. Reproduced from Experimental Biology and Medicine.^143^

Multiple studies have shown that inducing compressive stress and shear stress in cartilage and bone constructs respectively, generated a faster maturation in comparison to static conditions, as reviewed by Schulz and Bader^140^ and Yeatts and Fisher.^141^ Therefore, including these mechanical stimuli in osteochondral constructs could induce tissue development faster. The use of these bioreactors, Figure 8, could further be explored in the development of an OA disease model.

There are already bone and cartilage-based disease models that recreate certain aspects of OA through compressive loading,^143^ applying mechanical stress, and/or adding inflammatory cytokines.^144^ Young et al. aimed to produce specific stress and cell-based OA model using static and cyclic loading. This was performed using a compression device that would push against porcine chondrocytes encapsulated in a hydrogel cell carrier. They found that a static load of more than 40 psi applied for 24 h generated a decreased ECM anabolism, higher ECM degradation, and increased oxidative stress. They concluded that 60 psi of static loading would be sufficient to generate OA in chondrocytes and should be used to produce OA disease models in the future. Alternatively, Houtman et al. used osteochondral human explants and applied physiological loading or added inflammatory (IL-1β) or hypertrophy (triiodothyronine, T3) precursors; the latter were used to treat the explants for 6 days, while the mechanical compression was performed for 4 days at a strain of 65% with a frequency of 1 Hz. Although the three treatments showed changes in the explants related to OA, each one generated a different effect, showing that specific symptoms of OA can be recreated in vitro. Pro-inflammatory treatment showed the most severe cartilage breakdown, while the mechanical strain triggered OA-related changes via catabolism showing a cartilage ECM with abnormal elastic properties and water-retaining capabilities. Whereas the first one could be used to induce OA with more inflammatory characteristics, the second one could be used for mimicking OA that is produced post-traumatically. Despite the multiple approaches that OA disease modelling has taken with individual tissues or co-cultures, there is a lack of full bioprinted osteochondral tissues that mimic this disease. However, the use of bioreactors for faster maturation as well as for inducing OA should be explored to overcome current material limitations and further expand the field of 3D bioprinted disease models.

### Loss of long-term viability and communication between cartilage and bone: Vascularisation of the bone component

To develop a functional and representative osteochondral tissue, which could be used as an OA disease model where multiple stages of this disease can be recreated, the tissue construct must be kept viable for a prolonged period. This is also necessary to demonstrate the long-term effects of OA drugs and treatment strategies. An approach to maintaining osteochondral constructs viable for long periods maintaining the phenotype and functionality of both cartilage and bone would be to culture the constructs in a divided insert.^145^ Using such a culture system Kleuskens et al. demonstrated that different human osteochondral explants were kept viable and functional for as long as 4 weeks.^145^ However, this would only be feasible if the osteochondral constructs are small enough, so media diffusion is sufficient to reach its core.

Alternatively, an established vasculature system that keeps the bone component of the osteochondral unit viable and facilitates proper communication between the bone and the cartilage, could be necessary to ensure the long-term stability of the construct and its functionality. This approach would be the best when it comes to generating larger constructs in which diffusion is not enough to feed the central areas of the construct. Different techniques such as 3D micromolding,^146^ perfusion 3D bioprinted channels,^118^ and incorporation of additional cell types such as HUVECs^119^ to produce capillary-like structures have demonstrated their capability to generate functional vasculature in bone constructs. Recently, Chiesa et al. have successfully recreated a biomimetic bone model in vitro with robust vascularisation using endothelial cells via 3D bioprinting.^120^ Following up on these advances, the subsequent approach would be to combine these successful techniques used in the development of bone constructs with a chondral phase to bioengineer an osteochondral model.

### Incorporation of additional tissues for OA disease modelling

Further complexity could be included alongside the bioprinted osteochondral tissues to generate OA disease models, by incorporating additional tissue types. In this review, we have focused on the osteochondral unit. However, components, such as the synovial fluid and the synovial membrane, present in the joints, further add to the complex interplay between the different joint tissues. The inclusion of immune cells in the osteochondral joints, instead of direct cytokines, which represent an oversimplified approach for mimicking this complex disease, will perhaps recapitulate a closer model of the in vivo disease conditions. Previous 3D OA disease models have shown that OA characteristic symptoms can be also recreated by co-culturing chondrocytes and other relevant cells or tissues, such as macrophages or synoviocytes. For example, in 2016, Samavedi et al. developed a 3D chondrocyte-macrophage co-culture system to evaluate the interplay between activated murine macrophages and human chondrocytes in OA. They used both normal and osteoarthritic human chondrocytes encapsulated in poly(ethylene glycol) diacrylate (PEGDA) hydrogels. The co-culture system resulted in the development of a biomimetic tissue that more closely resembled in vivo scenarios than their respective mono-cultures.^136^ Additionally, Stellavato et al. have recently studied the anti-inflammatory effects of hybrid cooperative complexes (HCC) based on high and low molecular weight hyaluronan. They used an in vitro OA model based on human chondrocytes and synoviocytes. This co-culture model showed cellular responses that closely corresponded with OA symptoms observed in vivo, hence providing a reliable model to test the anti-inflammatory effects of HCC.^147^ If the primary co-culture principles could be integrated with the previously presented methods to develop OA in biofabricated osteochondral constructs, a better interplay between multiple components inherent to the human joint could be achieved.

Recreation of an in vitro OA disease model could be used in drug testing. For example, Lin et al. developed an osteochondral tissue chip derived from iPSCs using gelatine scaffolds. After chemically inducing OA-like inflammation, by adding IL-1β, they tested Celecoxib, a COX-2 (cyclooxygenase-2) inhibitor drug that downregulated the catabolic and pro-inflammatory cytokines present in the OA model.^148^ Although this was a biphasic construct with both cartilage and bone, which was able to recreate OA and show changes when applying drugs, it still had limitations in the osteochondral structure such as the tidemark, which was not observed. Once again, these 3D biofabricated osteochondral constructs may be useful in disease modelling investigations, as they could bring the necessary structural complexity.

**Figure 9.**
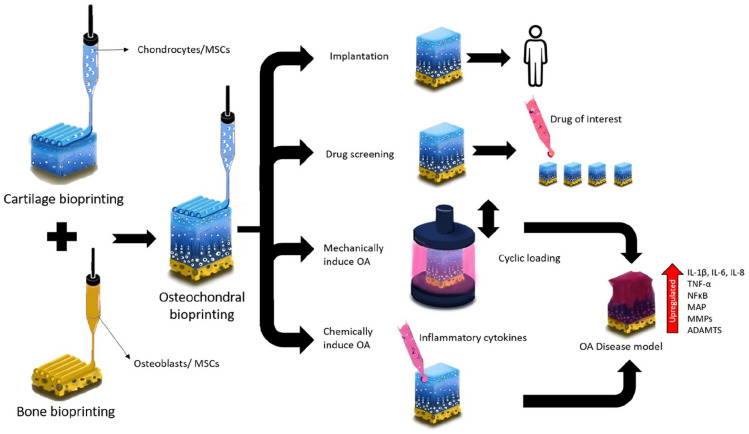
Proposed workflow of 3D bioprinted OA disease models. After bone and cartilage bioprinting, osteochondral bioprinted constructs can be used as implants, as platforms to perform high throughput drug screening, or subjected to mechanical loading or inflammatory cytokines to induce OA or other joint diseases in vitro.

In summary, Figure 9, 3D biofabrication techniques for osteochondral constructs must be enhanced to ensure long-term viability and mechanical fidelity to the physiological tissue. Once this is achieved, OA-like changes could be induced either mechanically or chemically to recreate its physiological changes and symptoms. Furthermore, these models could be used in personalised medicine to test specific drugs that could treat or control OA progression. This could be further developed by incorporating co-culture with additional cells/tissues, opening a new field of study in which the understanding of OA disease and the search for a cure would be accelerated.

## Conclusion

This review paper gives an overview of the state-of-the-art in 3D bioprinting of osteochondral tissues as a promising tool to develop physiologically representative osteochondral units. 3D bioprinting is a technique that may enable the production of osteochondral units to recreate disease models such as OA, osteochondral implants, or perform in vitro drug testing. The state-of-the-art shows that the most successful approaches to developing these tissues (bone, cartilage, and osteochondral) rely on the combination of cellular and acellular 3D bioprinting. However, vascularisation, the recreation of physiological characteristics, stability, and reproducibility of the bioprinted constructs, and the use of multiple tissues or cell types with an established communication network are important challenges and areas of development that still need to be resolved and further explored. Despite these challenges, the possible combinations of printing parameters, materials, cells, and GFs, using 3D bioprinting, in addition to the multiple potential strategies to improve the maturation and physiological characteristics of the constructs, bring on-demand human disease models, implants, and drug testing closer to reality.
